# The Arabidopsis *AMOT1/EIN3* gene plays an important role in the amelioration of ammonium toxicity

**DOI:** 10.1093/jxb/ery457

**Published:** 2019-01-24

**Authors:** Guangjie Li, Lin Zhang, Meng Wang, Dongwei Di, Herbert J Kronzucker, Weiming Shi

**Affiliations:** 1 State Key Laboratory of Soil and Sustainable Agriculture, Institute of Soil Science, Chinese Academy of Sciences, Nanjing, China; 2 University of the Chinese Academy of Sciences, Beijing, China; 3 School of Agriculture and Food, Faculty of Veterinary and Agricultural Sciences, The University of Melbourne, Parkville, VIC, Australia

**Keywords:** Ammonium stress, *amot1* mutant, Arabidopsis, AMOT1/EIN3, H_2_O_2_, peroxidases

## Abstract

Ammonium (NH_4_^+^) toxicity inhibits shoot growth in Arabidopsis, but the underlying mechanisms remain poorly characterized. Here, we show that a novel Arabidopsis mutant, *ammonium tolerance 1* (*amot1*), exhibits enhanced shoot growth tolerance to NH_4_^+^. Molecular cloning revealed that *amot1* is a new allele of *EIN3*, a key regulator of ethylene responses. The *amot1* mutant and the allelic *ein3-1* mutants show greater NH_4_^+^ tolerance than the wild type. Moreover, transgenic plants overexpressing *EIN3* (*EIN3ox*) are more sensitive to NH_4_^+^ toxicity The ethylene precursor 1-aminocyclopropane-1-carboxylic acid (ACC) increases shoot sensitivity to NH_4_^+^, whereas the ethylene perception inhibitor Ag^+^ decreases sensitivity. NH_4_^+^ induces ACC and ethylene accumulation. Furthermore, ethylene-insensitive mutants such as *etr1-3* and *ein3eil1* display enhanced NH_4_^+^ tolerance. In contrast, the ethylene overproduction mutant *eto1-1* exhibits decreased ammonium tolerance. AMOT1/EIN3 positively regulates shoot ROS accumulation, leading to oxidative stress under NH_4_^+^ stress, a trait that may be related to increased expression of peroxidase-encoding genes. These findings demonstrate the role of *AMOT1/EIN3* in NH_4_^+^ tolerance and confirm the strong link between NH_4_^+^ toxicity symptoms and the accumulation of hydrogen peroxide.

## Teaser

We identified a novel Arabidopsis *ammonium tolerance* 1 (*amot1*) mutant and reveal that blocking of ethylene signaling reduces tissue H_2_O_2_ accumulation and enhances seedling tolerance to ammonium stress.

## Introduction

Ammonium (NH_4_^+^), an important source of nitrogen for many species (Kronzucker *et al.*, 1997; Balkos *et al.*, 2010), is frequently present in soils and in the atmosphere in significant quantities (Britto and Kronzucker, 2002; Dupre *et al.*, 2009). However, NH_4_^+^ is toxic at moderate levels, frequently achieved in soils, to most plants, in particular those used in temperate agriculture, with stunted root and leaf growth as major symptoms of toxicity (Britto and Kronzucker, 2002; Coskun *et al.*, 2013). Several important physiological processes have been linked to excessive NH_4_^+^ exposure, such as ionic imbalance, relationships with carbon biochemistry, energy consumption, and modifications of hormonal balance (Britto and Kronzucker, 2002; Coskun *et al.*, 2013). Ethylene production has been shown to increase linearly with tissue NH_4_^+^ accumulation (Barker, 1999*a*), concurrent with the development of toxicity symptoms (You and Barker, 2002, 2005). In addition, the application of ethylene synthesis and ethylene action inhibitors can ameliorate NH_4_^+^ toxicity symptoms (Barker and Corey, 1991; Feng and Barker, 1992*a*, *b*; You and Barker, 2005; G. Li *et al.*, 2013). Ethylene is synthesized from *S*-adenosyl-l-methionine (SAM) via 1-aminocyclopropane-1-carboxylic acid (ACC). The rate-limiting step in ethylene biosynthesis lies in ACC production via ACC synthase (ACS), followed by ACC conversion to ethylene by ACC oxidase (ACO) (Adams and Yang, 1979). ACS and ACO are encoded by multigene families and are regulated by both developmental and environmental factors. Evolution of ethylene in response to biotic and abiotic stress resulting from ACS and ACO up-regulation is a common phenomenon (Yamagami *et al.*, 2003; Schellingen *et al.*, 2014). In Arabidopsis, the downstream component of the ethylene signaling pathway includes ethylene receptor ETRs (e.g. ETR1), CTR1 (constitutive triple response 1), EIN2 (ethylene-insensitive 2), EIN3/EIL (ethylene-insensitive 3/EIN3-like1), and ERF1 (ethylene-response factor 1) transcription factors (Lei *et al.*, 2011). Ethylene signaling is transduced into the nucleus to cause the accumulation of two master transcriptional activators EIN3 and EIL1, which initiate transcriptional re-programming in various ethylene responses (Yoo *et al.*, 2009; Tao *et al.*, 2015). However, the detailed mechanisms of ethylene biosynthesis and signaling in NH_4_^+^ stress responses remain unclear.

Reactive oxygen species (ROS) are induced under a wide range of environmental stresses; they can cause oxidative damage leading to injury and death, depending on cellular concentrations. NH_4_^+^ was found to induce higher levels of H_2_O_2_ and oxidative stress response reactions in leaves of the aquatic plant *Vallisneria natans* (Wang *et al.*, 2008), Arabidopsis (Podgórska *et al.*, 2013), and other species (Podgórska and Szal, 2015). However, data regarding the pathways involved in NH_4_^+^ regulation of H_2_O_2_ production are still rare. ROS can be generated in the apoplast via the activity of NADPH oxidases under stress (Mittler *et al.*, 2004). A group of NADPH oxidases and respiratory burst oxidase homologs (RBOHs) have been identified in Arabidopsis (Sagi and Fluhr, 2006). ROS are tightly regulated via a production/scavenging equilibrium. The expression of genes involved in the ROS regulatory network, including *APX1* (ascorbate peroxidase 1) and *CAT1* (catalase 1) in Arabidopsis (Davletova *et al.*, 2005; Xing *et al.*, 2008), also affects ROS levels. Peroxidases (PODs) have been proposed as alternative producers of ROS (Apel and Hirt, 2004; Bindschedler *et al.*, 2006). PODs catalyze the oxidoreduction of various substrates using H_2_O_2_. PODs, rather than NADPH oxidase, have been proposed as the major ROS producer in French bean (*Phaseolus vulgaris*) treated with a cell wall elicitor of *Colletotrichum lindemuthianum*, the fungus that causes anthracnose (Bolwell *et al.*, 1998). Kim *et al.* (2010) found that a POD contributes to ROS production during the Arabidopsis root response to potassium deficiency, showing the POD to be a component of the low potassium signal transduction pathway. Recently, Balzergue *et al.* (2017) showed that –Pi induces root tip ROS accumulation, indicating that PODs play a role. Further, the POD inhibitor salicylhydroxamic acid (SHAM) restored root growth and reduced ROS accumulation under –Pi conditions (Balzergue *et al.*, 2017). Although ethylene and ROS have been reported to be involved in NH_4_^+^ sensitivity, there has been no study to evaluate the role of ethylene in high-NH_4_^+^-induced ROS production in leaves, and further research is necessary to clarify the circumstances under which NH_4_^+^ causes oxidative stress in plants.

One approach to elucidating mechanisms of NH_4_^+^ toxicity in plants is to use mutant lines. Qin *et al.* (2008) isolated the first NH_4_^+^-sensitive root elongation mutant, *vtc1* (vitamin C defective 1), disrupted in GDP-mannose pyrophosphorylase (GMPase). Recently, several genetic regulators controlling root sensitivity to NH_4_^+^ have been identified in Arabidopsis, such as *aux1* (auxin resistant 1) (Cao *et al.*, 1993; Li *et al.*, 2011), *trh1* (tiny root hair 1) (Zou *et al.*, 2012), *dpms1* (dolichol phosphate mannose synthase1) (Jadid *et al.*, 2011), and *gsa1* (gravitropism sensitive to ammonium1) (Zou *et al.*, 2013). Elucidation of the function of these genetic regulators in determining the root sensitivity to NH_4_^+^ offered insight into the molecular basis of historically described physiological responses to NH_4_^+^ toxicity. In contrast, the underlying mechanisms of impaired leaf growth under NH_4_^+^ toxicity are still largely unknown. Reduced shoot biomass and leaf chlorosis are important symptoms (Britto and Kronzucker, 2002). Based on their chlorotic phenotypes, ammonium-overly-sensitive 1 (*amos1*) (B. Li *et al.*, 2012) and *amos2* (G. Li *et al.*, 2012) mutants were recently identified: the *AMOS1* locus is identical to *EGY1* (ethylene-dependent gravitropism-deficient and yellow-green-like protein1), which encodes a membrane-bound, ATP-independent metalloprotease localized to plastids, required for chloroplast biogenesis (Chen *et al.*, 2005). However, the genetic locus responsible for the *amos2* mutation has not been identified. These studies, in combination, provide a significantly improved understanding of the process of NH_4_^+^ toxicity in plants.

Here, we report a novel *Arabidopsis thaliana* mutant, *amot1* (ammonium tolerance 1), which displays enhanced shoot growth in response to NH_4_^+^ stress. Gene cloning shows *amot1* to be allelic to *EIN3*. Our results demonstrate that the disruption of AMOT1/EIN3 reduces high-NH_4_^+^-induced ROS accumulation in leaves, leading to reduced oxidative stress in the shoot. Moreover, AMOT1/EIN3 up-regulates shoot expression of the genes coding for PODs, previously shown to correlate positively with NH_4_^+^-induced changes in ROS content and cell growth inhibition.

## Materials and methods

### Plant materials and growth conditions

Plant materials used in this work included wild-type (WT) *A. thaliana* L. (Col-0 ecotype) and genetic mutants derived from the Col-0 background. The mutants *ein3-1* (Chao *et al.*, 1997), *eil1-1* (Alonso *et al.*, 2003), *ein3-1eil1-1* (Alonso *et al.*, 2003), *EIN3ox* (*35S:EIN3*) (Chao *et al.*, 1997), and *5×EBS:GUS*/Col-0 transgenic plants (He *et al.*, 2011) were described previously. The *eto1-1* (Alonso and Stepanova, 2004), *etr1-3* (Guzmán and Ecker, 1990), and *rbohd* mutants were obtained from the Arabidopsis Biological Resource Center (ABRC). Seeds were surface-sterilized and cold-treated at 4 °C for 48 h prior to being sown onto standard growth medium. The standard growth medium was described previously (G. Li *et al.*, 2013) and was composed of 2 mM KH_2_PO_4_, 5 mM NaNO_3_, 2 mM MgSO_4_, 1 mM CaCl_2_, 0.1 mM Fe-EDTA, 50 μM H_3_BO_3_, 12 μM MnSO_4_, 1 μM ZnCl_2_, 1 μM CuSO_4_, 0.2 μM Na_2_MoO_4_, 0.5 g l^–1^ MES, 1% sucrose, and 0.8% agarose (pH 5.7, adjusted with 1 M NaOH). The day of sowing was considered day 0. Seedlings were grown, oriented vertically on the surface of the medium in a growth chamber, under a 16 h light/8 h dark photoperiod, an irradiance of 100 μmol m^−2^ s^−1^, and a constant temperature of 23±1 °C. Other chemical treatments were provided as additions to the growth medium, as indicated.

### Screening conditions

Transfer DNA (T-DNA) lines were constructed in the laboratories of D. Weigel and C. Somerville using the pSKI15 vector. Approximately 7500 independent lines (stock no. N21995) were provided by the ABRC. After surface sterilization, seeds were sown and grown on vertically oriented growth medium plates. After 5 d, seedlings were transferred to growth medium plates supplemented with 20 mM (NH_4_)_2_SO_4_. Potential NH_4_^+^ tolerance mutants were selected after 6 d and rescued, transferred to soil, and allowed to self-fertilize. The homozygous M_4_*amot1* mutant was backcrossed to the WT Col-0, and the resulting F_1_ generation was crossed with WT Col-0 twice to remove unlinked mutations caused by the mutagenesis.

### Thermal asymmetric interlaced PCR

DNA for PCR amplification was extracted according to Weigel and Glazebrook (2002). Plant T-DNA-flanking sequences were amplified by PCR according to the protocols of Rodrigues *et al.* (2009).

The following primers were used: SKI1, 5'-AATTGGTAATTACTCTTTCTTTTCCTCCATATTGA-3'; SKI2, 5'-ATATTGACCATCATACTCATTGCTGATCCAT-3'; SKI3, 5'-TGATCCATGTAGATTTCCCGGACATGAA-3'; AD1, 5'-TG(AT)G(ACGT)AG(GC)A(ACGT)CA(GC)AGA-3'; AD2, 5'-(ACGT)TCGA(GC)T(AT)T(GC)G(AT)GTT-3'; AD3, 5'-(ACGT)GTCGA(GC)(AT)GA(ACGT)A(AT)GAA-3'; AD4, 5'-AG(AT)-G(ACGT)AG(AT)A(ACGT)CA(AT)AGG-3'; AD5, 5'-(AT)GTG(ACGT)AG-(AT)A(ACGT)CA(ACGT)AGA-3'; and AD6, 5'-(GC)TTG(ACGT)TA(GC)T-(ACGT)CT(ACGT)TGC-3'.

### Growth assays

For high-NH_4_^+^ stress experiments, 5-day-old seedlings were transferred onto growth medium containing various concentrations of (NH_4_)_2_SO_4_. Following 6 d of treatment, photographs were taken, and relative rosette size and shoot biomass were measured. To study the effect of precursors or inhibitors, the medium was supplemented with NH_4_^+^ plus the indicated concentrations of ACC (Sigma), AgNO_3_ (Shanghai yuanye biotechnology Co. Ltd, Shanghai, China), H_2_O_2_ (Shanghai yuanye biotechnology Co. Ltd), or SHAM (Shanghai yuanye biotechnology Co. Ltd). The ratio of average rosette size on NH_4_^+^-stressed plates to the average rosette size on control plates was calculated as relative rosette size, according to Lei *et al.* (2011). The fresh weight of each individual shoot was measured immediately after harvest using a high-precision balance (0.000001) (XP105, Mettler Toledo).

### NH_4_^+^, H_2_O_2_, MDA, and ACC content, and peroxidase and glutamine synthetase activity assay

Shoots (30–50 mg FW) of each sample were washed with 10 mM CaSO_4_, frozen in liquid nitrogen, and then extracted with 1 ml of 10 mM formic acid for the NH_4_^+^ content assay by HPLC, following derivatization with *o*-phthaldialdehyde (Sigma) as described previously (G. Li *et al.*, 2012). H_2_O_2_ content was determined by the POD-coupled assay according to Veljovic-Jovanovic *et al.* (2002). Arabidopsis shoots were ground in liquid nitrogen, and the powder was extracted in 2 ml of 1 M HClO_4_ in the presence of insoluble polyvinylpyrrolidone (5%). The homogenate was centrifuged at 12 000 *g* for 10 min, and the supernatant was neutralized with 5 M K_2_CO_3_ to pH 5.6 in the presence of 100 ml of 0.3 M phosphate buffer (pH 5.6). The solution was centrifuged at 12 000 *g* for 1 min, and the sample was incubated for 10 min with 1 U of ascorbate oxidase (Shanghai yuanye biotechnology Co. Ltd) to oxidize ascorbate prior to use in the assay. The reaction mixture consisted of 0.1 M phosphate buffer (pH 6.5), 3.3 mM DMAB (Shanghai yuanye biotechnology Co. Ltd), 0.07 mM MBTH (Shanghai yuanye biotechnology Co. Ltd), and 0.3 U of POD (Shanghai yuanye biotechnology Co. Ltd). The reaction was initiated by the addition of the sample. The absorbance change at 590 nm was monitored at 25 °C. The malondialdehyde (MDA) level was measured using a thiobarbituric acid-reactive substance (TBARS) assay kit (Nanjing Jiancheng Bioengineering Institute, Nanjing, China) (Ren *et al.*, 2015). Shoot ACC content was detected by negative ion chemical ionization (NICI) GC-MS (Schellingen *et al.*, 2014). Data are expressed in nmol g^–1^ FW. POD activity was detected by a micro-POD assay kit (BC0095, Solarbio, Beijing, China) according to the manufacturer’s recommendations. POD activity is expressed as U mg^–1^ based on protein content. Glutamine synthetase (GS) activity was detected by a GS kit (BC0910, Solarbio) (Zhao *et al.*, 2017). The specific enzyme activity (U g^–1^ FW) was defined as the amount of enzyme units catalyzing the transformation of 1 μM substrate per minute by the amount of fresh weight in grams.

### Real-time quantitative PCR analysis

Total RNA was extracted from Arabidopsis shoots. Gene sequences were provided by the National Center of Biotechnology Information (NCBI), and gene-specific primers for real-time quantitative PCR (qRT-PCR) were designed using Primer-5 software (see Supplementary Table S1 at *JXB* online). *CBP20* (nuclear-encoded cap-binding protein) and *ACTIN2* were used as the internal reference genes, and relative RNA abundance was normalized to the *CBP20* or *ACTIN2* internal control ([mRNA]_gene_/[mRNA]_CBP20_ or [mRNA]_gene_/[mRNA]_ACTIN2_).

### Histochemical staining and image analysis

Histochemical staining of H_2_O_2_ was performed as previously described (Dong *et al.*, 2009) with minor modiﬁcations. Shoots were vacuum-inﬁltrated with 0.1 mg ml^–1^ 3,3′-diaminobenzidine (DAB) in 50 mM Tris-acetate buffer, at pH 5.0. Samples were incubated for 24 h at room temperature in the dark prior to transfer to 80% ethanol. Histochemical analysis of β-glucuronidase (GUS) reporter enzyme activity was performed as described by Weigel and Glazebrook (2002). GUS or H_2_O_2_ staining in the shoot was carried out using an Olympus SZX10 stereo microscope. Intensities of the GUS- and DAB-stained zone were quantified using Image-J software. All staining and image analysis procedures were repeated at least twice.

### Ethylene measurements

After seedling exposure to 40 mM NH_4_^+^ for varying durations, as indicated, shoots from the control and treatments were weighed and put into 5 ml gas-tight vials containing 1 ml of agar medium (0.7% agar). Headspace samples (1 ml) were withdrawn and analyzed using a GC-6850 gas chromatograph (Agilent Technologies Japan, Ltd), which was equipped with an FID detector.

### Yeast one-hybrid (Y1H) analysis

Promoter fragments from At1g49570 (2217 bp) and At5g19890 (1692 bp) were cloned into the pAbAi vector to produce the bait constructs pAbAi-At1g49570 and pAbAi-At5g19890, respectively. The coding seuqence (CDS) of *AMOT1/EIN3* was fused to the pGADT7 vector to generate a prey construct, AD-*EIN3*. The bait construct and the empty vector (AD) served as the negative control; p53-AbAi/pGAD-p53 were used as a positive control and transformed separately into yeast cells. Transformed yeast cells were diluted with a 10× dilution series and dotted onto SD plates lacking Ura and Leu (with or without antibiotic).

### Statistical and graphical analyses

For all experiments, data were statistically analyzed using the SPSS 13.0 program (SPSS Chicago, IL, USA). Details are shown in the figure legends. Graphs were produced using Origin 8.0. All graphs and images were arranged using Adobe Photoshop 7.0.

## Results

### Enhanced tolerance of the *amot1* mutant to ammonium toxicity

Under our growth conditions, NH_4_^+^, at concentrations of 20–30 mM, caused slight reductions in shoot size and biomass (Supplementary Fig. S1). A concentration of 40 mM significantly inhibited shoot size and biomass (Supplementary Fig. S1). To explore the mechanisms of NH_4_^+^-induced shoot growth inhibition, we performed a forward genetic screen for seedlings that show a shoot phenotype that was more resistant than WT plants when grown on medium containing 40 mM NH_4_^+^. Seedlings that appeared similar to the WT without NH_4_^+^ but displayed significantly higher resistance of shoot growth to NH_4_^+^ were *amot* (ammonium tolerance) mutants. We present the characterization of the *amot1* mutant.

**Fig. 1.**
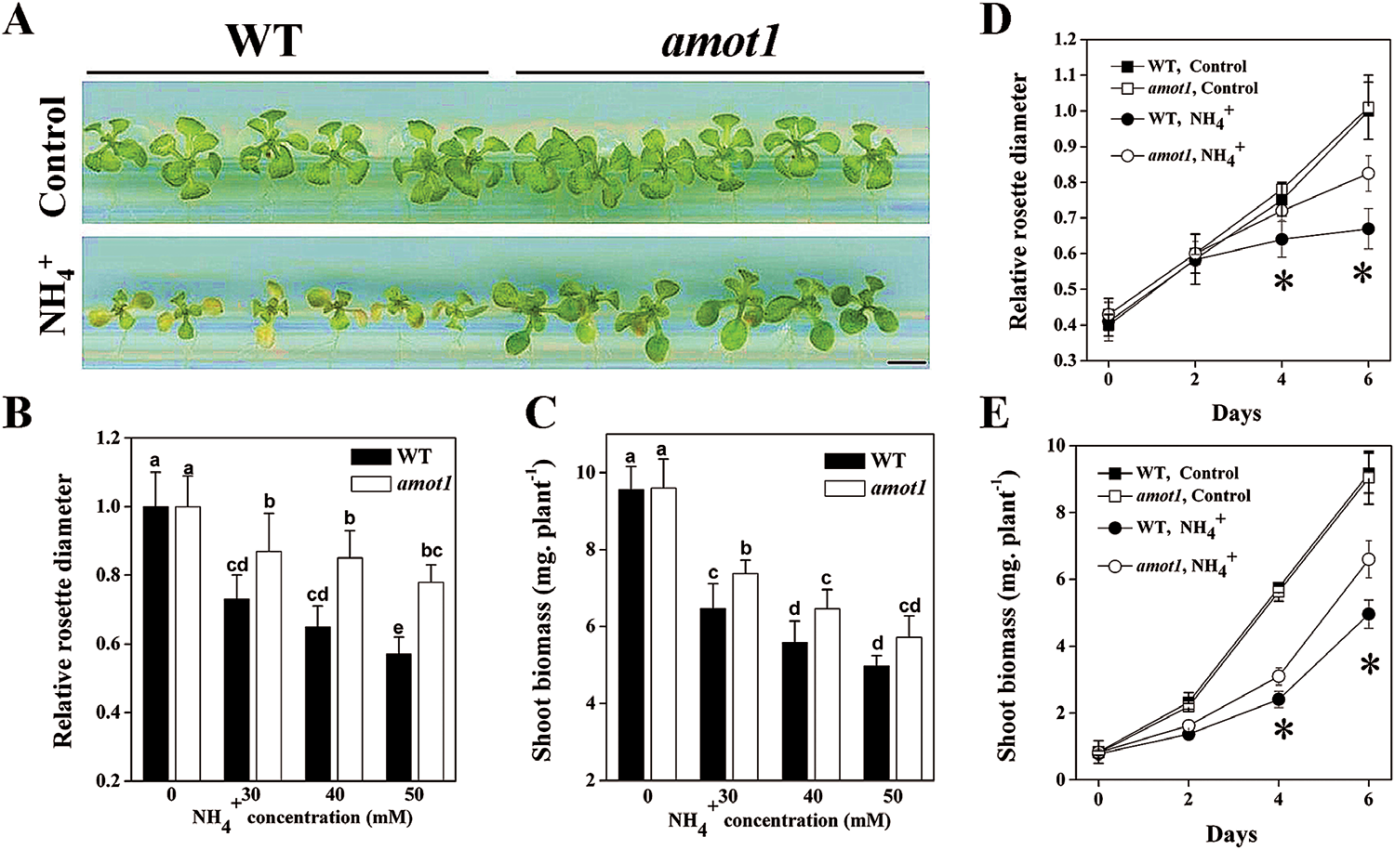
Isolation and characterization of the ammonium-tolerant *amot1* mutant. (A) Appearance of *Arabidopsis thaliana* wild-type (WT) and *amot1* mutant plants following treatment with NH_4_^+^. Five-day-old plants were transferred to control and 40 mM NH_4_^+^ concentration for 6 d, and then pictures were taken. Scale bars=0.5 cm. (B and C) Relative rosette diameter and fresh shoot weight of *A. thaliana* WT and *amot1* mutant plants following treatment with various NH_4_^+^ concentrations for 6 d. The rosette diameter on control nutrient solution was considered as 1. Values are the means ±SD, *n*=8–11. Different letters indicate statistical differences at *P*<0.05 (one-way ANOVA with Duncan post-hoc test). (D and E) Relative rosette diameter and fresh shoot weight of *A. thaliana* WT and *amot1* mutant plants following treatment with 40 mM NH_4_^+^ for 0, 2, 4, and 6 d. Values are the means ±SD, *n*=6–10. Asterisks indicate statistical differences between the WT and *amot1* under NH_4_^+^ treatment at the indicated times (independent samples *t*-test, *P<0.05). (This figure is available in color at *JXB* online.)

In agar plates without NH_4_^+^, the *amot1* shoot growth phenotype was indistinguishable from that of the WT (Fig. 1A). However, when grown in the presence of high NH_4_^+^, *amot1* shoot growth displayed greater resistance to NH_4_^+^ than the WT (Fig. 1A).

NH_4_^+^-treated WT and *amot1* plants showed a dose-dependent inhibitory effect of NH_4_^+^ on the growth of aerial parts in response to a range of NH_4_^+^ concentrations, but WT shoot growth was inhibited more than in *amot1* at the concentrations used (Fig. 1B, C). Furthermore, we analyzed the shoot phenotypes of *amot1* and WT seedlings in response to 40 mM NH_4_^+^ over time. The shoot growth between WT and *amot1* seedlings remained similar 2 d after NH_4_^+^ addition, but the difference was clearly accentuated under prolonged NH_4_^+^ treatment, with the mutant maintaining significantly higher growth rates (Fig. 1D, E). Considering these results together, *amot1* emerges as the first NH_4_^+^-resistant mutant, displaying superior shoot growth.

**Fig. 2.**
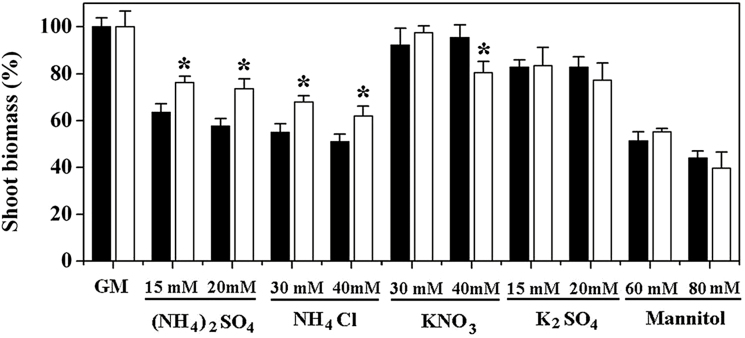
Specificity of the *amot1* mutant to NH_4_^+^. WT and *amot1* seedlings were grown for 5 d on growth medium (GM) and then transferred to medium supplemented with salts and osmotica as indicated. Shoot fresh weight was measured 6 d after transfer. Growth on control nutrient (GM) was considered as 100%. Values are the means ±SD, *n*=10–14. Asterisks indicate statistical differences between the mutant and WT (independent samples *t*-test, *P<0.05).

The WT and *amot1* seedlings were also treated on medium enriched with a variety of ions and molecules, and the results indicate that *amot1* seedlings are highly resistant to both (NH_4_)_2_SO_4_ and NH_4_Cl, but responded to 15 mM and 20 mM K_2_SO_4_ or 60 mM and 80 mM mannitol in a similar pattern to the WT (Fig. 2).

### The *amot1* mutant is a novel *ein3* allele, and loss of *EIN3* function enhances ammonium tolerance

**Fig. 3.**
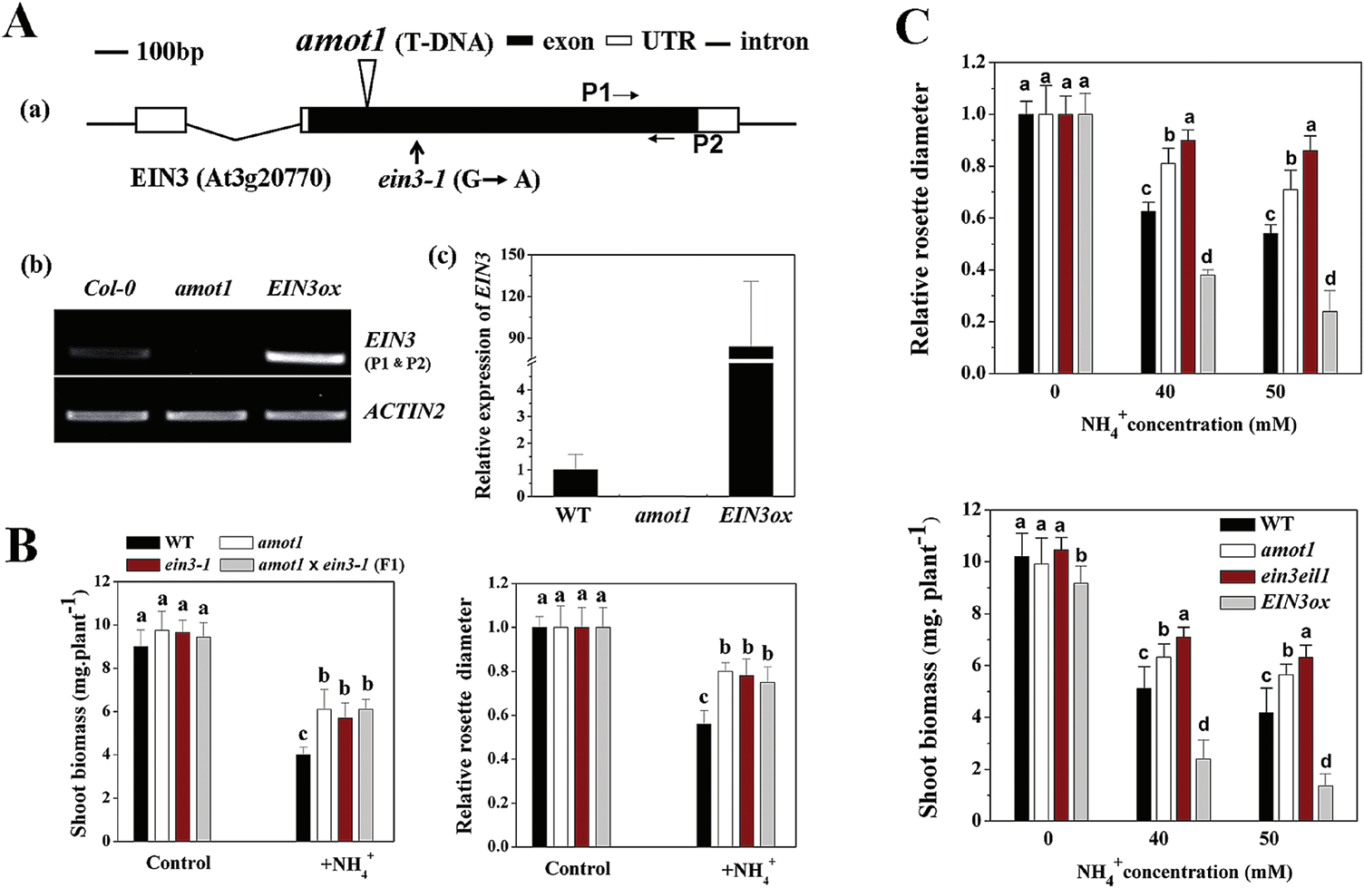
Molecular characterization of the *Arabidopsis thaliana amot1* mutant. (A) (a) Diagram illustrating the genomic coding sequence of the Arabidopsis *AMOT1/EIN3* gene and the locations of the mutant alleles *amot1* and *ein3-1*. UTR, untranslated region. (b) RT-PCR analysis of *EIN3* transcripts in WT, *EIN3ox*, and the *amot1* mutant plants. The *ACTIN2* gene was used as an internal control. (c) Expression of *EIN3* in WT, *EIN3ox*, and the *amot1* mutant plants analyzed by qRT-PCR analysis. Values are means ±SD of three replicates. *ACTIN2* was used as the internal reference gene, and *EIN3* expression of the WT was considered as 1. (B) *amot1* is allelic to the *ein3-1* mutant. WT, *amot1*, *ein3-1*, and F_1_ progeny from crosses between *amot1* and *ein3-1* were grown on 40 mM NH_4_^+^ for 6 d. The relative rosette diameter on control nutrient solution was considered as 1. Values are the means ±SD, *n*=7–12. (C) Effect of various NH_4_^+^ concentrations on relative rosette diameter, and fresh shoot weight of WT, *ein3eil1*, *amot1*, and *EIN3ox* seedlings. Five-day-old plants were transferred to control and 40 mM NH_4_^+^ concentration for 6 d. Values are the means ±SD, *n*=5–8. Different letters indicate statistical differences at *P*<0.05 (one-way ANOVA analysis with Duncan post-hoc test). (This figure is available in color at *JXB* online.)

The WT as female parent was crossed with the homozygous mutant as the pollen donor. F_1_ plants were selfed to obtain F_2_ seeds. Both F_1_ and F_2_ seeds were assayed for growth on NH_4_^+^ medium. All examined F_1_ progeny (45 seedlings) displayed the same phenotypes as the WT. In the F_2_ population, the *amot1* phenotype segregated at an approximate 1:3 ratio (*amot1*:WT=54:142; *x*^*2*^=0.55, *P*>0.05), indicating that *amot1* is a recessive mutation at a single nuclear locus. T-DNA-flanking sequences were isolated from the mutant by thermal asymmetric interlaced PCR, and sequence analysis revealed that the pSKI15 T-DNA was inserted into the exon of the *EIN3* gene (At3g20770) in *amot1*, 192 bp downstream of the start codon, ATG [Fig. 3A(a)]. *EIN3* gene transcripts were greatly reduced in *amot1* compared with the WT [Fig. 3A(b) and (c)]. To ascertain further whether the NH_4_^+^-resistant phenotype in *amot1* is due to the mutation in the *EIN3* gene, we analyzed the previously reported allele *ein3-1* (Chao *et al.*, 1997) and crossed the *amot1* mutant with *ein3-1* plants. With exposure to 40 mM NH_4_^+^, shoot size and biomass in *ein3-1* seedlings were indeed similar to those of *amot1* seedlings (Fig. 3B). Furthermore, the *amot1* mutant was crossed to *ein3-1*, and the F_1_ progeny showed a phenotype similar to that of the parents in the presence of NH_4_^+^ (Fig. 3B). Collectively, these results show that the *amot1* mutant is a new loss-of-function allele of the *EIN3* gene.

AMOT1/EIN3 is a member of a protein family that includes EIN3-like (EIL) proteins (Chao *et al.*, 1997) and initiates transcriptional re-programming in various ethylene responses (Guo and Ecker, 2004; Peng *et al.*, 2014). We sought to determine the role of AMOT1/EIN3 in NH_4_^+^ resistance. Because AMOT1/EIN3 and its close homolog EIL1 functionally overlap (Chao *et al.*, 1997; An *et al.*, 2010), we examined the *ein3-1eil1-1* double mutant seedling response under various NH_4_^+^ concentrations. Under high NH_4_^+^, *ein3-1eil1-1* had superior tolerance to NH_4_^+^ compared with the WT (26% reduction in *ein3-1eil1-1* versus 52% in the WT at 40 mM NH_4_^+^) (Fig. 3C). Furthermore, in contrast to the *ein3* single mutant’s tolerant phenotype, the *ein3-1eil1-1* double mutant exhibited a more tolerant phenotype than the *amot1* mutant (Fig. 3C). We also examined the phenotype of the *eil1* single mutant upon treatment with high NH_4_^+^; however, the *eil1-1* mutant was similar to the WT under high NH_4_^+^ (Supplementary Fig. S2). Next, we examined the NH_4_^+^-responsive phenotype of a transgenic line overexpressing EIN3 under the control of the 35S promoter (35S:EIN3/*Col-0* or *EIN3ox*), which displays an enhanced ethylene response (Chao *et al.*, 1997; An *et al.*, 2010; Z. Li *et al.*, 2013). The transcripts of *AMOT1/EIN3* were significantly increased in the *EIN3ox* seedlings [Z. Li *et al.*, 2013; Fig. 3A(b) and (c)]. Under high NH_4_^+^, *EIN3ox* plants displayed increased sensitivity, based on shoot size and biomass, when compared with their WT counterparts (73% shoot biomass reduction in *EIN3ox* plants versus 52% in the WT at 40 mM NH_4_^+^) (Fig. 3C). Together, these results suggest that constitutive overexpression of *AMOT1/EIN3* leads to elevated shoot NH_4_^+^ sensitivity in Arabidopsis. Consistent with a previous report (G. Li *et al.*, 2013), the *ein3eil1* lateral root number was also more resistant than that of the WT to high-NH_4_^+^ stress (Supplementary Fig. S3).

### Enhanced shoot ethylene evolution is involved in ammonium-mediated inhibition of shoot growth

**Fig. 4.**
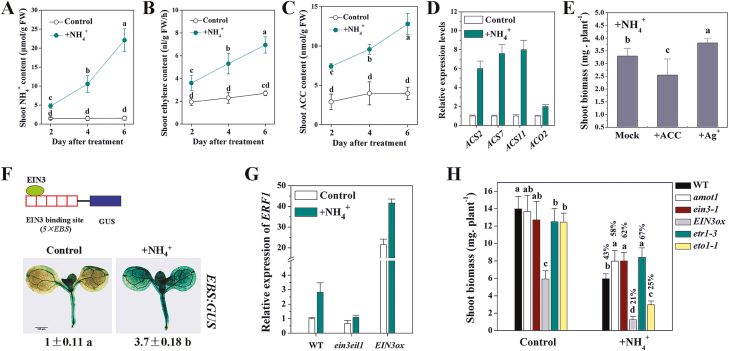
Effects of ethylene on shoot growth tolerance to NH_4_^+^. (A) NH_4_^+^ content in Arabidopsis shoots for the duration of the NH_4_^+^ treatment. (B) Ethylene evolution in Arabidopsis shoots for the duration of the NH_4_^+^ treatment. (C) 1-Aminocyclopropane-1-carboxylic acid (ACC) content in Arabidopsis shoots for the duration of the NH_4_^+^ treatment. Seedlings at 5 d after germination were exposed to NH_4_^+^ for varying treatment times, and NH_4_^+^ content (A), ethylene evolution (B), and ACC content were determined. Values are means ±SD of three replicates. Different letters indicate statistical differences at *P*<0.05 (one-way ANOVA with Duncan post-hoc test). (D) Effect of NH_4_^+^ treatment on shoot *ACS* and *ACO* genes expression by qRT-PCR for 6 h. Values are means ±SD of three replicates. *CBP20* was used as the internal reference gene, and the control was considered as 1. (E) Effect of supplied ethylene inhibitors 30 μM AgNO_3_ and 25 μM ACC on shoot biomass of WT seedlings grown in 40 mM NH_4_^+^ treatment medium. Values are the means ±SD, *n*=10–12. (F) Schematic diagram of the EIN3 activity reporter system showing the EIN3 protein, five tandem repeats of the EBS (*5×EBS*), and the *GUS* gene. Expression of *5×EBS:GUS* in leaves of the WT under control conditions and 24 h NH_4_^+^ treatment. One representative sample from each treatment (10 plants) is shown. GUS staining intensity was quantified using Image J software, and the control was considered as 1. Values are means ±SD of three replicates. (G) Effect of NH_4_^+^ treatment on shoot *ERF1* gene expression of WT, *ein3eil1*, and *EIN3ox* lines by qRT-PCR for 6 h. Values are means ±SD of three replicates. *ACTIN2* was used as the internal reference gene, and the WT control was considered as 1. (H) Effect of NH_4_^+^ treatment for 6 d on shoot fresh weight of WT, *amot1*, *ein3-1*, *EIN3ox*, *etr1-3*, and *eto1-1* seedlings. Values are the means ±SD, *n*=12. Different letters indicate statistical differences at *P*<0.05 of control and NH_4_^+^ treatment, respectively (one-way ANOVA with Duncan post-hoc test).

Aerial tissue NH_4_^+^ content was determined, and the NH_4_^+^ content increased gradually with treatment time compared with that in untreated shoots (Fig. 4A). Shoot ethylene production under NH_4_^+^ exposure was also significantly greater than without NH_4_^+^ and increased linearly with NH_4_^+^ treatment time (Fig. 4B), consistent with Barker (1999*b*). Ethylene is synthesized from SAM via ACC, which is catalyzed by the enzyme ACS (Adams and Yang, 1979). ACC amounts in untreated and treated seedlings are presented in Fig. 4C. Consistent with previous reports, ACC amounts increased linearly with NH_4_^+^ treatment time. As ACS and ACO are key enzymes of the ethylene biosynthetic pathway in plants, *AtACS2*, *AtACS7*, *AtACS11*, and *AtACO2* expression was examined. Expression of the four genes was rapidly up-regulated in response to high NH_4_^+^ (Fig. 4D).

We further investigated the activity of AMOT1/EIN3 in response to NH_4_^+^ in shoot. A transgenic reporter line harboring the GUS gene driven by five tandem repeats of the EIN3-binding site (EBS) followed by a minimal 35S promoter (*5×EBS:GUS*/Col-0) has been used to monitor the transcriptional activity of EIN3 (Stepanova *et al.*, 2007; He *et al.*, 2011). Following NH_4_^+^ treatment, GUS staining became intensified in the cotyledons of *5×EBS:GUS*/Col-0 plants (Fig. 4F), indicative of elevated levels of AMOT1/EIN3 activity. We also observed that the expression of the ethylene-responsive gene *ERF1* was up-regulated by NH_4_^+^ in the WT (Fig. 4G). In keeping with the results on AMOT1/EIN3 activity, expression of *ERF1*, a direct target gene of EIN3 (Solano *et al.*, 1998), was also lower in the NH_4_^+^-treated *ein3eil1* mutant, but higher in *EIN3ox* lines, compared with the WT (Fig. 4G).

The WT plants treated with the ethylene biosynthetic precursor ACC displayed decreased tolerance to NH_4_^+^ (Fig. 4E). Consistent with this, the ethylene overproduction mutant *eto1-1* (ethylene overproducer 1) also showed reduced NH_4_^+^ tolerance compared with the WT (Fig. 4H). In the presence of the ethylene receptor antagonist Ag^+^, shoot growth of the WT was significantly increased when the plants were exposed to NH_4_^+^ stress (Fig. 4E). As ethylene is known to activate downstream signaling pathways by binding to ethylene receptors (e.g. ETR1), we examined whether ethylene regulates shoot growth sensitivity to NH_4_^+^ in such a way. Shoot growth in the ethylene-insensitive (ethylene receptor) mutant *etr1-3* and positive regulator mutants in ethylene signaling, *amot1* and *ein3-1*, was more tolerant to NH_4_^+^ than that of the WT; consistent with this, *EIN3ox* lines displayed increased shoot growth sensitivity (Fig. 4H). These results indicate that ethylene has a negative effect on NH_4_^+^ tolerance in Arabidopsis shoot growth.

### AMOT1/EIN3 regulates ammonium-induced ROS accumulation in shoots

**Fig. 5.**
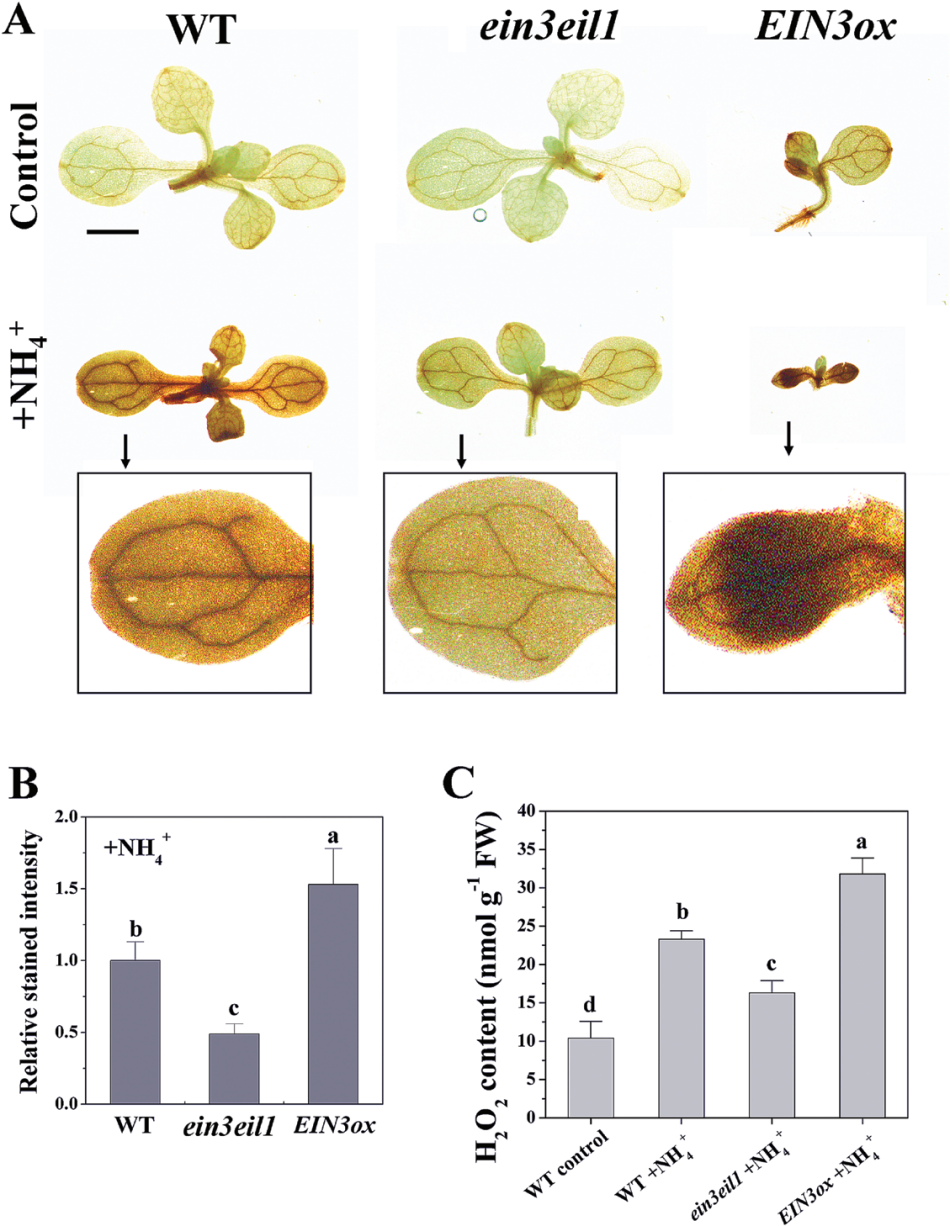
Effects of EIN3 on NH_4_^+^-induced H_2_O_2_ accumulation in shoots. (A) *In situ* detection of WT, *ein3eil1*, and *EIN3ox* leaf H_2_O_2_. Seedlings at 5 d were exposed to 40 mM NH_4_^+^ for 3 d, and then DAB staining of shoots was performed. Scale bars=1 mm. The inserts show images of partial enlargement, as indicated by arrows. (B) The mean relative DAB staining intensity in the WT, *ein3eil1*, and *EIN3ox* of the NH_4_^+^-treated shoots in (A), and the WT was considered as 1. Values are the means ±SD, *n*=10–15. (C) H_2_O_2_ concentration in the WT, *EIN3ox*, and *ein3eil1* shoot tissue. Seedlings at 5 d were exposed to 40 mM NH_4_^+^ for 3 d, and the contents of H_2_O_2_ were determined as described in the Materials and methods. Values are means ±SD of three replicates. Different letters indicate statistical differences at *P*<0.05 (one-way ANOVA with Duncan post-hoc test).

High NH_4_^+^ induces an increase in ROS in plants; however, the biological mechanism of NH_4_^+^-induced ROS accumulation remains largely unknown. Here, we examined the levels of endogenous H_2_O_2_ in the WT, *ein3eil1*, and *EIN3ox* in response to high-NH_4_^+^ treatment. NH_4_^+^ stress increased H_2_O_2_ accumulation in the cotyledons of the WT, indicated by DAB staining (Fig. 5A, B). We further found higher levels of DAB staining in *EIN3ox* but lower levels in *ein3eil1* than in the WT following NH_4_^+^ stress (Fig. 5A, B), in accordance with the NH_4_^+^ tolerance phenotypes of these genotypes (Fig. 3C). We also measured the H_2_O_2_ contents in shoots. As inferred from DAB staining assays, high-NH_4_^+^ treatment induced accumulation of H_2_O_2_ in the WT. However, the level was lower in *ein3eil1* and higher in *EIN3ox* shoots compared with Col-0 under NH_4_^+^ stress (Fig. 5C). The H_2_O_2_ content under control conditions was not significantly different among the three ecotypes (Supplementary Fig. S4). A split-shoot experiment was devised to examine further the relationship between NH_4_^+^-induced AMOT1/EIN3 transcriptional activity and ROS accumulation (Supplementary Fig. S5A). The cotyledon supplied with NH_4_^+^ displayed significantly increased *EBS:GUS* expression compared with the untreated cotyledon (Supplementary Fig. S5B). Consistent with *EBS:GUS* inductive sites, a higher intensity of DAB staining was also detected in the NH_4_^+^-treated cotyledon (Supplementary Fig. S5C).

**Fig. 6.**
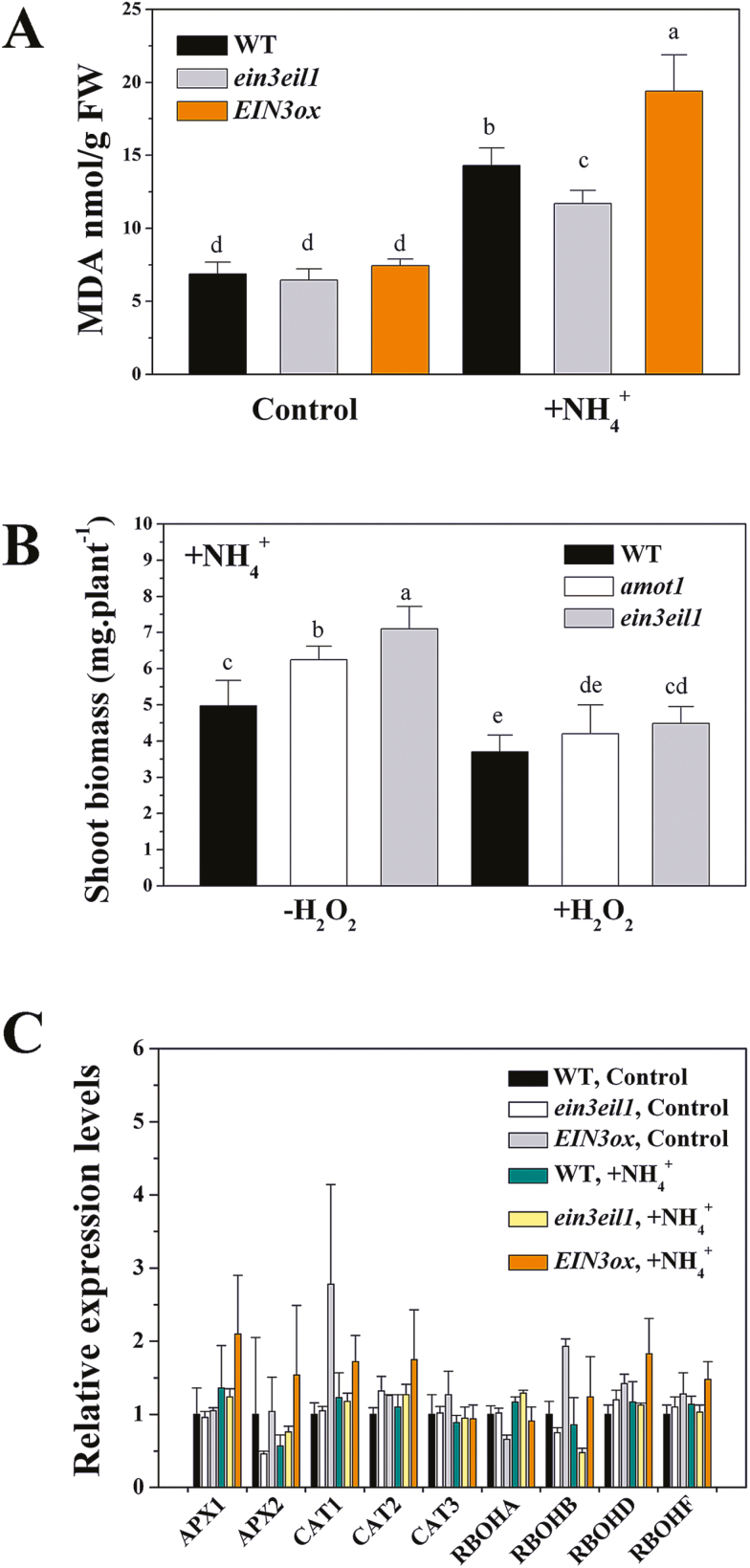
Effects of EIN3 on NH_4_^+^-induced lipid peroxidation in shoots and related gene expression. (A) Lipid peroxidation (MDA content) in the WT, *ein3eil1*, and *EIN3ox* shoot tissue. Seedlings at 5 d were exposed to 40 mM NH_4_^+^ for 6 d. Values are means ±SD of three replicates. (B) Effect of external H_2_O_2_ on shoot biomass of WT, *ein3eil1*, and *amot1* plants under NH_4_^+^ treatment. Five-day-old seedlings were transferred to medium supplemented with NH_4_^+^ alone or in combination with 2 mM H_2_O_2_ for 6 d. *n*=7–10. (C) Effect of NH_4_^+^ on gene expression of WT, *EIN3ox*, and *ein3eil1* shoot tissue by qRT-PCR for 6 h. Values are means ±SD of three replicates. *ACTIN2* was used as the internal reference gene, and the WT control was considered as 1. Different letters indicate statistical differences at *P*<0.05 (one-way ANOVA with Duncan post-hoc test).

In this study, the concentration of MDA equivalents was increased in NH_4_^+^-treated leaves (Fig. 6A). However, we observed a higher MDA level in *EIN3ox*, and a lower level in *ein3eil1* than in the WT following NH_4_^+^ stress (Fig. 6A). The effects of NH_4_^+^ on shoot growth were also examined in combination with external H_2_O_2_. The combined treatment with NH_4_^+^ and H_2_O_2_ in the medium markedly inhibited shoot growth compared with NH_4_^+^ alone, and the combined treatment inhibited shoot growth more significantly in *amot1* and *ein3eil1* (Fig. 6B).

We further examined the expression of genes encoding antioxidant metabolic enzymes, such as *APX1*, *APX2*, *CAT1*, *CAT2*, and *CAT3*. NH_4_^+^ stress did not induce *APX* and *CAT* gene expression in WT shoots, and these genes were also not much affected in *ein3eil1* and *EIN3ox* seedlings under NH_4_^+^ stress (Fig. 6C), suggesting that AMOT1/EIN3 regulation of NH_4_^+^-induced ROS accumulation might not be related to *APX1-*, *APX2-*, *CAT1-*, *CAT2-*, and *CAT3*-mediated antioxidant activity. Previous studies showed that drought stress increases *RBOH* transcript levels (Lee *et al.*, 2012). However, the expression patterns of RBOHA, RBOHB, RBOHD, and RBOHF under control and high-NH_4_^+^ stress were similar, and they were also not much affected in the *ein3eil1* mutant and in *EIN3ox* lines compared with the WT (Fig. 6C). Furthermore, DAB staining and shoot growth in response to high-NH_4_^+^ stress were also similar between the WT and the *rbohd* mutant (Supplementary Fig. S6).

### AMOT1/EIN3 induces the transcription of peroxidases and increases their activity

**Fig. 7.**
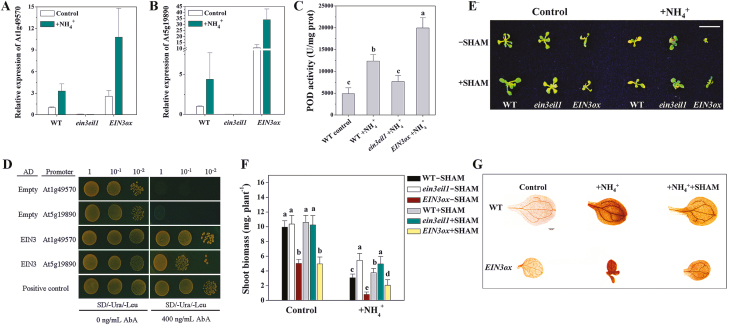
EIN3 increases activity of PODs through transcriptional regulation of POD genes. (A and B) qRT-PCR analysis of the expression of two POD genes (At5g19890 and At1g48570) in WT, *EIN3ox*, and *ein3eil1* shoot tissue after NH_4_^+^ treatment for 6 h. Values are means ±SD of three replicates. *ACTIN2* was used as the internal reference gene, and the WT control was considered as 1. (C) Measurement of POD activity of WT, *EIN3ox*, and *ein3eil1* shoot tissue. Seedlings were exposed to 40 mM NH_4_^+^ for 5 d. Values are means ±SD of three replicates. (D) Y1H assay showing the EIN3 binding to the promoter of the two POD genes. The yeast expression plasmid pGADT7-EIN3 was reintroduced into the yeast strain Y1H Gold carrying the pAbAi-At1g49570 or pAbAi-At5g19890 vectors. The transformants (with or without dilutions) were screened for their growth on yeast synthetic defined medium (SD/-Ura -Leu) in the presence of 400 ng ml^–1^ AbA (antibiotic) for stringent selection. The empty vectors pGADT7 and p53-AbAi/pGAD-p53 were used as a negative and positive control, respectively. (E and F) Effect of salicylhydroxamic acid (SHAM) on the shoot biomass of the WT, *EIN3ox*, and *ein3eil1*. Five-day-old seedlings were transferred to medium alone or in combination with 10 μM SHAM for 6 d. A photograph of representative seedlings is shown in (E). Scale bars=1 cm. The shoot biomass is shown in (F). Values are the means ±SD, *n*=12. Different letters indicate statistical differences at *P*<0.05 of control and NH_4_^+^ treatment, respectively (one-way ANOVA with Duncan post-hoc test). (G) Effect of SHAM on the NH_4_^+^-induced H_2_O_2_ accumulation in shoots of WT and *EIN3ox*. Seedlings at 5 d were exposed to 40 mM NH_4_^+^ with or without 10 μM SHAM for 3 d, and then DAB staining of shoots was performed. Scale bars=200 μm.

Podgórska *et al.* (2015) proposed that higher POD levels are positively correlated with NH_4_^+^-induced ROS generation and cell growth inhibition. We found that the expression of two of the genes encoding PODs, At5g19890 and At1g48570, was induced by NH_4_^+^ treatment in the WT, and expression was more elevated in *EIN3ox* while it was reduced in *ein3eil1* with or without NH_4_^+^ (Fig. 7A, B). The transcript levels of other POD-encoding genes, such as At5g42180, At2g18980, At4g11290, and At3g49960, were not increased by NH_4_^+^ treatment in the WT, and were also not significantly altered in *EIN3ox* and *ein3eil1* seedlings, under either normal or NH_4_^+^ stress conditions (Supplementary Fig. S7). The POD activity assay also showed that POD activity was significantly elevated in *EIN3ox* seedlings compared with the WT and *ein3eil1* under NH_4_^+^ stress (Fig. 7C). POD activity in *EIN3ox* seedlings under control conditions was also slightly elevated compared with the WT and with *ein3eil1* (Supplementary Fig. S8). We next analyzed the promoter regions of two POD genes (At5g19890 and At1g48570) and found EBSs (ATGTA) in each promoter (data not shown). To test the interaction between the AMOT1/EIN3 protein and the At5g19890 and At1g48570 promoters, a Y1H assay was performed. As shown in Fig. 7D, bait yeast cells co-transformed with the empty vector (AD) or the fusion vector (AD-EIN3) grew well on synthetic dropout medium (SD) without Ura and Leu. However, only the yeast cells co-transformed with the fusion vector AD-EIN3 survive on the selective medium supplemented with 400 ng ml^–1^ aureobasidin A (AbA; Fig. 7D). The data suggest that the AMOT1/EIN3 protein interacts with the At5g19890 and At1g48570 promoters in the yeast system. SHAM is a widely used POD inhibitor (Balzergue *et al.*, 2017). The shoot biomass of the WT and *EIN3ox* lines was increased under NH_4_^+^ stress after SHAM treatment, but no effects on *ein3eil1* lines were observed (Fig. 7E, F). Furthermore, the POD inhibitor SHAM could decrease NH_4_^+^-induced DAB staining, indicating H_2_O_2_ accumulation in both WT and *EIN3ox* leaves (Fig. 7G; Supplementary Fig. S9).

### The *amot1* mutant accumulates less NH_4_^+^ in shoot tissue under NH_4_^+^ toxicity

**Fig. 8.**
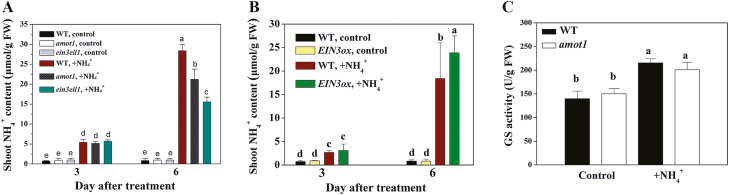
Effects of NH_4_^+^ treatment on shoot NH_4_^+^ content and GS activity. (A) NH_4_^+^ contents in the shoot tissues of WT, *ein3eil1*, and *amot1* seedlings. Five-day-old WT, *ein3eil1*, and *amot1* seedlings were grown on growth medium and transferred to fresh medium with control or NH_4_^+^ for 3 d and 6 d of growth, and then NH_4_^+^ tissue content was determined. Values are means ±SD of three replicates. (B) NH_4_^+^ contents in the shoot tissues of WT and *EIN3ox* seedlings. Five-day-old WT and *EIN3ox* seedlings were grown on growth medium and transferred to fresh medium with control or NH_4_^+^ for 3 d and 6 d of growth, and then NH_4_^+^ tissue content was determined. Values are means ±SD of three replicates. (C) GS activities in the shoots of WT and *amot1* seedlings. Five-day-old WT and *amot1* seedlings were grown on growth medium and transferred to fresh medium with control or NH_4_^+^ for 6 d, and then GS activity was determined. Values are means ±SD of three replicates. Different letters indicate statistical differences at *P<*0.05 (one-way ANOVA with Duncan post-hoc test).

There was no difference in shoot NH_4_^+^ content of the WT, *amot1*, and *ein3eil1* with 3 d of NH_4_^+^ treatment (Fig. 8A), although ROS accumulation was greater in WT shoots than in those of *ein3eil1* at this treatment time (Fig. 5). However, higher levels of NH_4_^+^ in the WT, but lower levels in *amot1* and *ein3eil1*, than in the WT were recorded following high-NH_4_^+^ stress during a 6 d treatment (Fig. 8A). Consistently, NH_4_^+^ accumulation was slightly higher in *EIN3ox* shoots than in the WT following the treatment (Fig. 8B). Activities of the enzyme GS, centrally involved in the NH_4_^+^ assimilation process (Kronzucker *et al.*, 1995; Hirano *et al.*, 2008), was determined in shoots of the WT and *amot1*. GS activity was induced by external NH_4_^+^ in NH_4_^+^-fed WT and *amot1* plants, but this increase was not significantly different in the two genotypes (Fig.8C). This indicates that NH_4_^+^ metabolism was not affected by the *AMOT1* mutation.

## Discussion

Stunted root system and decreased leaf bomass are among the most visible phenotypic manifestations of NH_4_^+^ toxicity in higher plants (Britto and Kronzucker, 2002). Several genetic regulators controlling root sensitivity to NH_4_^+^ have been identified in Arabidopsis (Li *et al.*, 2014); however, little is known about the specific targets and pathways that lead to impaired leaf growth in the context of NH_4_^+^ toxicity. To gain insight into the mechanisms of the effects of NH_4_^+^ on shoot growth, we employed a molecular genetics approach, based on a mutant screen for altered response to NH_4_^+^. In the current work, enhanced NH_4_^+^ tolerance of shoot growth was found in *amot1*. We further revealed that the nuclear *AMOT1* locus is identical to *EIN3*, which encodes a transcriptional activator required for initiating transcriptional re-programming in various ethylene responses (Guo and Ecker, 2004; Yoo *et al.*, 2009). It was found that *amot1* and *ein3-1*, a reported allele, showed enhanced shoot growth tolerance compared with the WT, but the transgenic line overexpressing EIN3 (*EIN3ox*) was more sensitive. The activity of AMOT1/EIN3, indicated by using *EBS:GUS* in shoots, was markedly enhanced on NH_4_^+^ (Fig. 4F). These results suggest that AMOT1/EIN3 plays an important role in the NH_4_^+^-induced impairment of shoot growth. It was demonstrated furthermore that this inhibitory effect is related to enhanced shoot ACC and ethylene accumulation. More importantly, it was found that AMOT1/EIN3 positively regulates shoot ROS accumulation, which leads to oxidative stress in Arabidopsis shoots under NH_4_^+^ stress, and up-regulates the shoot expression of the genes coding for PODs previously shown to correlate positively with NH_4_^+^-induced increases in ROS content and cell growth inhibition (Podgórska *et al.*, 2015).

A role for ethylene evolution has long been suggested under NH_4_^+^ excess (Barker and Corey, 1991; Barker, 1999*a*, *b*), but its involvement remains incompletely understood. The present data indicate that ethylene evolution increases linearly with NH_4_^+^ treatment time (Fig. 4B), consistent with previous reports (Barker 1999*b*). Foliar ethylene evolution increased sharply in tomato when foliar NH_4_^+^ accumulation passed a critical value (Barker, 1999*a*). Our present and previous data also link the stimulation of *EBS:GUS* activity to NH_4_^+^ exposure (G. Li *et al.*, 2013; Supplementary Fig. S5). The present data show that shoot NH_4_^+^ content increases linearly with increased treatment time (Fig. 4A). Furthermore, a previous observation showed high ethylene evolution to correlate with high tissue NH_4_^+^ but to be independent of nitrogen form and pH regime (Feng and Barker, 1992*c*). Hence, together with previous reports, our study suggests that greatly increased shoot NH_4_^+^ content may be the intrinsic trigger leading to enhanced ethylene evolution under NH_4_^+^ stress. The rate-limiting step in ethylene biosynthesis lies in the production of ACC by ACS (Schellingen *et al.*, 2014). Shoot ACC amounts also increased linearly with NH_4_^+^ treatment time (Fig. 4C). Furthermore, we show here that *AtACS2*, *AtACS7*, *AtACS11*, and *AtACO2*, which encode ACS and ACO, the two key enzymes responsible for ethylene synthesis, are transcriptionally up-regulated by NH_4_^+^ treatment (Fig. 4D). Therefore, it is conceivable that the increased ACC biosynthesis resulting from up-regulation of ACS and ACO gene expression is involved in NH_4_^+^-induced ethylene evolution. Further study on the detailed mechanisms of NH_4_^+^-regulated ethylene evolution is warranted.

In our study, we provide several lines of evidence supporting the notion that ethylene biosynthesis and signaling play a negative role in the adaptation of Arabidopsis shoot growth to NH_4_^+^ stress. ACC content, ethylene production, and AMOT1/EIN3 activity, and the expression of genes encoding key enzymes responsible for ethylene synthesis in the Arabidopsis shoot showed dramatic increases after NH_4_^+^ treatment. The dual evidence that shoot biomass was inhibited by NH_4_^+^ to a greater extent in the ethylene overproduction mutant *eto1-1* and the *EIN3ox* line, compared with the WT, and that mutations in ethylene receptors (e.g. *etr1-3*) and key positive regulators in ethylene signaling (e.g. *amot1* and *ein3-1*) showed increased shoot growth compared with the WT under NH_4_^+^ stress (Figs 3, 4H), supports this notion. The observations that the externally supplied ethylene inhibitor Ag^+^ alleviated the phenotypic manifestation of toxicity, but that ACC, a precursor of ethylene, aggravated NH_4_^+^-suppressed shoot growth in the WT (Fig. 4E), further demonstrate the important role of shoot ethylene signaling. Our findings are in excellent agreement with a previous study showing that Ag^+^ improved plant growth on NH_4_^+^ (Barker and Corey, 1991). Therefore, the data suggest that ethylene biosynthesis and signaling negatively regulate NH_4_^+^ tolerance of shoot growth in Arabidopsis.

The underlying mechanisms determining ROS accumulation by NH_4_^+^ in leaves are only partially understood (Bittsánszky *et al.*, 2015). Our results show that AMOT1/EIN3 is involved in H_2_O_2_ metabolism in leaves under NH_4_^+^ stress. First, a higher level of H_2_O_2_ cytochemical staining in *EIN3ox* was found, while a lower level of H_2_O_2_ staining was seen in *ein3eil1* than in the WT following NH_4_^+^ stress (Fig. 5A, B), in accordance with the NH_4_^+^ tolerance phenotypes and lipid peroxidation profiles of these genotypes (Figs 3, 6A). In agreement with the above results, NH_4_^+^ stress increased leaf H_2_O_2_ concentrations in the WT, while these were lower in *ein3eil1* and higher in *EIN3ox* under identical treatment conditions (Fig. 5C). Moreover, our split-shoot experiment showed that a higher DAB staining intensity was detected in the components of *EBS:GUS* cotyledons exposed to NH_4_^+^ (Supplementary Fig. S5). These results suggest that AMOT1/EIN3 positively regulates NH_4_^+^-induced leaf H_2_O_2_ accumulation. Investigations on whether oxidative stress is involved in NH_4_^+^ phytotoxicity have led to conflicting conclusions. The results of Domínguez-Valdivia *et al.* (2008) in spinach and pea suggest that stress originating from applying NH_4_^+^ as the only nitrogen source is not an oxidative stress. However, evidence is accumulating that NH_4_^+^ can induce oxidative stress in leaves, including in Arabidopsis (Podgórska and Szal, 2015), the aquatic plant *Hydrilla verticillata* (Wang *et al.*, 2010), and duckweed (Huang *et al.*, 2013). Our present results show that externally supplied H_2_O_2_ increases shoot growth sensitivity of *amot1* and *ein3eil1* to NH_4_^+^ stress (Fig. 6B). The extent of lipid peroxidation, estimated by monitoring the decomposition product MDA, has also been reported as elevated in NH_4_^+^-grown seedlings (Hachiya *et al.*, 2010; Podgórska *et al.*, 2013; Fig. 6A). Together, these findings indicate that increased H_2_O_2_ accumulation and oxidative stress in leaves under NH_4_^+^ stress at least partially result from elevated shoot AMOT1/EIN3 activity, providing a molecular basis for NH_4_^+^-induced accumulation of H_2_O_2_ in leaves. However, the present study shows that, although lower, there was still increased H_2_O_2_ accumulation and oxidative stress in *ein3eil1* mutant leaves under NH_4_^+^ stress (Figs 5, 6A), suggesting that there are pathways through which AMOT1/EIN3 functions independently to regulate shoot H_2_O_2_ accumulation and oxidative stress in response to NH_4_^+^ stress.

Drought can promote ROS biosynthesis by inducing expression of several *Atrboh* genes, such as *AtrbohA*, *AtrbohD*, and *AtrbohE* (Lee *et al.*, 2012). However, Podgórska *et al.* (2015) found that expression of the RBOHD gene is not induced in leaves under NH_4_^+^ stress. In this study, we show that there is no increase in RBOHA, RBOHB, RBOHD, or RBOHF expression in NH_4_^+^-treated WT, *EIN3ox*, and *ein3eil1* leaves. These results suggest that AMOT1/EIN3 is not involved through modulating expression of *Atrboh* genes under high NH_4_^+^, such as RBOHA, RBOHB, RBOHD, and RBOHF. However, we cannot exclude NADPH oxidase as a potential ROS source in NH_4_^+^-treated plants, as other RBOH genes might be up-regulated. NH_4_^+^-mediated changes in apoplastic pH (Husted and Schjoerring, 1995) may induce ROS generation, possibly through the modulation of the activities of cell wall PODs (Lager *et al.*, 2010). Furthermore, under low-Pi conditions, increased POD activities also inhibited Arabidopsis root growth by regulating ROS levels and cell wall stiffening, and the POD inhibitor SHAM could restore root growth and reduce ROS accumulation under –Pi (Balzergue *et al.*, 2017). Podgórska *et al.* (2015) showed that higher POD levels positively correlate with NH_4_^+^-induced ROS content and cell growth inhibition. Consistent with this, by examining POD gene expression and POD activity under NH_4_^+^ conditions, we show that NH_4_^+^ stress increases expression of some POD genes (At5g19890 and At1g48570) and POD activity in WT shoots (Fig. 7A–C). Moreover, the peroxidase inhibitor SHAM could indeed alleviate NH_4_^+^-induced shoot ROS accumulation (Fig. 7G; Supplementary Fig. S9) and growth inhibition (Fig. 7E, F). This result, together with previous reports, confirms that NH_4_^+^ induces accumulation of ROS and suggests that POD expression and activity may play an important role. Our qRT-PCR analyses show that two POD genes (At5g19890 and At1g48570) were constitutively up-regulated in *EIN3ox* but down-regulated in the *ein3eil1* double mutant, regardless of NH_4_^+^ (Fig. 7A). Moreover, NH_4_^+^-induced POD activity was positively correlated with the expression of EIN3 genes, as shown for *ein3eil1* and *EIN3ox* seedlings (Fig. 7B). Further studies revealed that the key transcription factor AMOT1/EIN3 may directly target the two POD genes (At5g19890 and At1g48570), as the AMOT1/EIN3 protein could specifically bind to the promoters of the At5g19890 and At1g48570 genes, as revealed by the Y1H assay. Further supporting our finding of increased POD activity and DAB staining, indicating H_2_O_2_ accumulation in *EIN3ox*, the POD inhibitor SHAM was shown to enhance *EIN3ox* shoot growth and reduce DAB staining used to indicate H_2_O_2_ accumulation under NH_4_^+^ stress (Fig. 7; Supplementary Fig. S9). Collectively, these data indicate that the ethylene signaling-mediated NH_4_^+^ response of Arabidopsis shoot growth is brought about, at least partially, through POD genes (e.g. At5g19890 and At1g48570), via AMOT1/EIN3.

The accumulation of free shoot NH_4_^+^ is widely considered to be critical to the development of NH_4_^+^ toxicity (Gerendas *et al.*, 1997; Szczerba *et al.*, 2008). Because H_2_O_2_ accumulation was greater in WT shoots than in *ein3eil1*, but higher in *EIN3ox* than in the WT, after a 3 d treatment with high NH_4_^+^, we hypothesized that loss of function or overexpression of AMOT1/EIN3 may entail reduced or enhanced NH_4_^+^ content in the shoot at the 3 d treatment time, respectively. However, there was no difference in shoot NH_4_^+^ content between the WT, *amot1*, *ein3eil1*, and *EIN3ox* (Fig. 8A). These results further highlight that AMOT1/EIN3 plays an important role in regulating NH_4_^+^-induced shoot ROS accumulation and rules out that reduced shoot ROS accumulation during the early phase of exposure (within 3 d in our study) by loss of function of AMOT1/EIN3 resulted from reduced NH_4_^+^ content. However, a higher NH_4_^+^ content in *EIN3ox* and the WT, and a lower content in *amot1* and *ein3eil1* were observed following high-NH_4_^+^ stress for prolonged treatment times (6 d) (Fig. 8A, B). Our results indicate that GS activity was not affected by the mutation in *AMOT1*/*EIN3* (Fig. 8C), showing that GS is not responsible for the lower NH_4_^+^ accumulation in the *amot1* mutant. Alternatively, oxidative stress itself, induced by H_2_O_2_, can increase cellular NH_4_^+^ concentrations by inducing proteolytic activity (Sweetlove *et al.*, 2002). It is not clear how AMOT1/EIN3 mediates shoot NH_4_^+^ accumulation over longer periods of treatment, and more research is warranted to examine this.

**Fig. 9.**
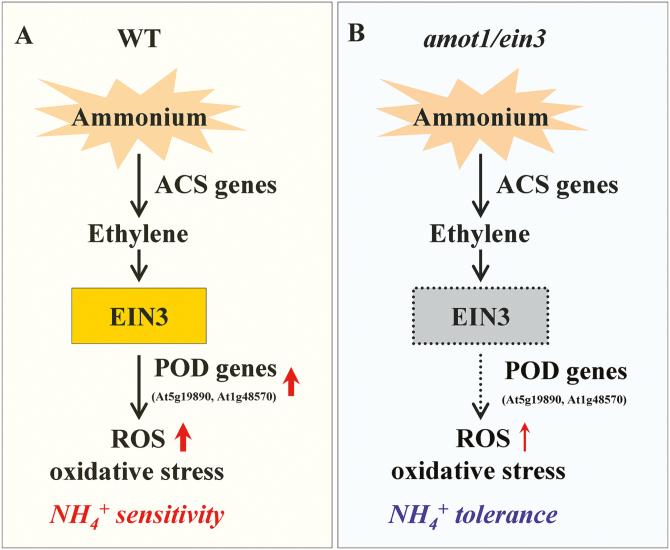
A proposed model for ethylene–EIN3 function in shoot NH_4_^+^ sensitivity. Based on our study and previous reports (Chao *et al.*, 1997; G. Li *et al.*, 2013; Podgorska *et al.*, 2013, 2015), we established a model for ethylene–EIN3 function in shoot NH_4_^+^ sensitivity. (A) In the wild type, NH_4_^+^ stress enhanced the expression of *ACS* and *ACO* genes, encoding ACS and ACO, the two key enzymes responsible for ethylene synthesis. Under NH_4_^+^ stress, ethylene is perceived and transduced, affecting the transcription factor EIN3, and initiating the ethylene response. EIN3 regulates ROS accumulation, which leads to oxidative stress in Arabidopsis shoots under NH_4_^+^ stress. The expression of EIN3-mediated POD genes (e.g. At5g19890 and At1g48570) is involved in NH_4_^+^ stress-induced shoot ROS accumulation. (B) In the *amot1/ein3* mutant, expression of the AMOT1/EIN3-dependent POD genes (e.g. At5g19890 and At1g48570) in the shoot is blocked under NH_4_^+^ stress. Ethylene regulation of ROS accumulation and oxidative stress is lowered. Orthogons in orange represent known EIN3 functions, and orthogons in gray with dashed lines represent the inhibition of EIN3 functions due to the *amot1/ein3* mutation. Red arrows indicate increased POD gene expression, ROS accumulation, and oxidative stress, and thick and thin red arrows indicate, respectively, a high or low ROS accumulation and oxidative stress.

In summary, we have identified and characterized a novel gene, *AMOT1*/*EIN3*, that controls shoot NH_4_^+^ sensitivity and propose a model for ethylene–AMOT1/EIN3 functions in shoot NH_4_^+^ sensitivity (Fig. 9). Our study shows the importance of EIN3 in shoot inhibition under high-NH_4_^+^ stress, providing strong genetic evidence in support of the role of the ethylene biosynthesis and signaling pathway in regulating shoot NH_4_^+^ sensitivity. Under NH_4_^+^ stress, ethylene is perceived and transduced, affecting the transcription factors EIN3/EIL and initiating the ethylene response. Additionally, our work highlights the roles of AMOT1/EIN3 in regulating ROS accumulation in Arabidopsis shoots under NH_4_^+^ stress. In the *ein3eil1* mutant, high-NH_4_^+^-induced ROS accumulation is reduced, which leads to reduced oxidative stress in the shoot. However, *EIN3ox* shoots accumulated more ROS and displayed higher sensitivity to NH_4_^+^ stress. Elucidation of this interaction between AMOT1/EIN3 and H_2_O_2_ signaling provides novel insight into our understanding on how EIN3 regulates shoot growth under high-NH_4_^+^ stress. Moreover, it was found that AMOT1/EIN3 can up-regulate shoot expression of the genes coding for PODs (e.g. At5g19890 and At1g48570) previously shown to correlate positively with NH_4_^+^-induced ROS accumulation and cell growth inhibition. Further studies using molecular approaches to investigate the transcriptional network regulated by AMOT1/EIN3 will provide important new insights into the process of acclimation and of adaptation to NH_4_^+^ stress in plants.

## Supplementary data

Supplementary data are available at *JXB* online.

Fig. S1. Rosette diameter and fresh shoot weight of *Arabidopsis thaliana* wild-type (WT, Col-0) plants following treatment with various NH_4_^+^ concentrations.

Fig. S2. Relative rosette diameter (A) and fresh shoot weight (B) of *Arabidopsis thaliana* WT and *eil1* mutant plants following treatment with NH_4_^+^ for 6 d.

Fig. S3. Lateral root number of *Arabidopsis thaliana* WT and *ein3eil1* mutant plants following treatment with high NH_4_^+^ for 6 d.

Fig. S4. H_2_O_2_ content in WT, *EIN3ox*, and *ein3eil1* shoot tissue under control conditions.

Fig. S5. *EBS:GUS* expression and DAB staining in the split-shoot experiment.

Fig. S6. Effect of NH_4_^+^ treatment on shoot DAB staining and biomass of the WT and the *Atrbohd* mutant.

Fig. S7. qRT-PCR analysis of POD gene expression in WT, *EIN3ox*, and *ein3eil1* shoot tissue under NH_4_^+^ treatment for 6 h.

Fig. S8. Measurement of POD activity of WT, *EIN3ox*, and *ein3eil1* shoot tissue under control conditions.

Fig. S9. Mean relative DAB staining intensity in WT (A) and *EIN3ox* (B) shoots treated with NH_4_^+^ and NH_4_^+^ plus SHAM.

Table S1. Gene-specific primers used for qRT-PCR.

Supplementary Material
