# Supplementary material for: The Arabidopsis AMOT1/EIN3 gene plays an important role in the amelioration of ammonium toxicity
Source: J Exp Bot. 2019 Jan 24;70(4):1375–88. doi: 10.1093/jxb/ery457 (PMC6382331; doi:10.1093/jxb/ery457)
Supplement: Supplementary Material [file ery457_suppl_supplementary_material.pdf]

The *Arabidopsis AMOT1/EIN3* gene plays an important role in the amelioration of ammonium toxicity

Guangjie Li, Lin Zhang, Meng Wang, Dongwei Di, Herbert J. Kronzucker, Weiming Shi

Supplementary Data

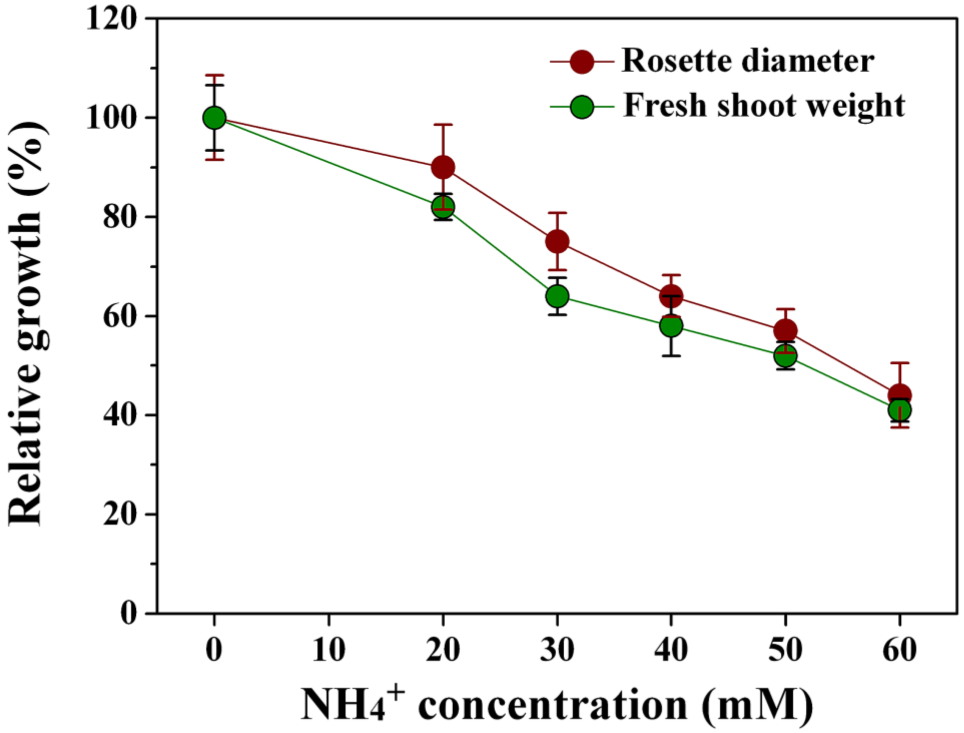

Fig. S1 Rosette diameter and fresh shoot weight of *Arabidopsis thaliana* wild-type (WT, *Col-0*) plants following treatment with various NH_4^+ concentrations. 5-d-old plants were transferred to solutions containing the indicated NH_4^+ concentrations for 6 d, and then rosette diameter and fresh shoot weight were measured. Growth on control nutrient solution was considered as 100%. Values are the means ± SD, n = 10–12.

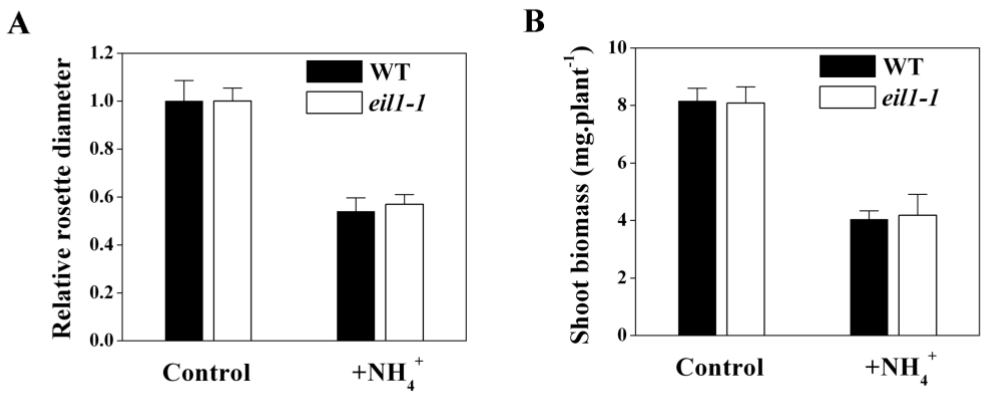

Fig. S2 Relative rosette diameter (A) and fresh shoot weight (B) of *Arabidopsis thaliana* WT and *eil1* mutant plants following treatment with NH_4^+ for 6 d. Rosette diameter on control nutrient solution was considered as 1. Values are the means ± SD, n = 6-8.

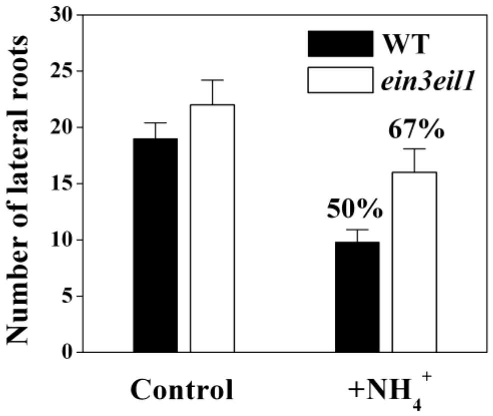

Fig. S3 Lateral root number of *Arabidopsis thaliana* WT and *ein3eil1* mutant plants following treatment with high NH_4^+ for 6 d. Values are the means ± SD, n = 8-10.

Fig. S4 H_2O_2 content in WT, *EIN3ox,* and *ein3eil1* shoot tissue under control conditions. Seedlings at 5 d were exposed to control medium without NH_4^+ for 3 d, and the contents of H_2O_2 were determined as described in Materials and Methods. Values are means ± SD of three replicates. FW, fresh weight. Different letters indicate statistical differences at $P < 0.05$ (one-way ANOVA analysis with Duncan post-hoc test).

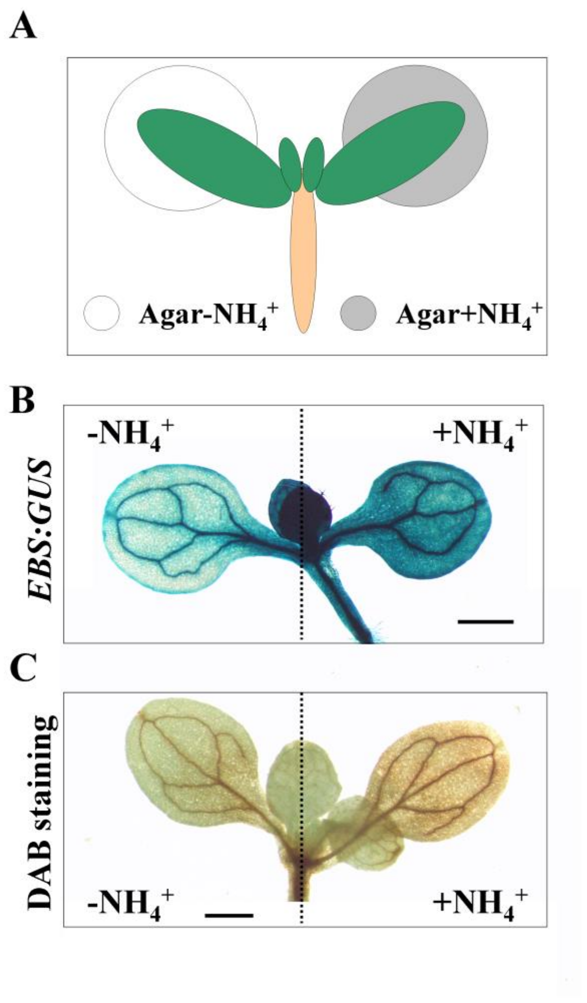

Fig. S5 *EBS:GUS* expression and DAB staining in the split-shoot experiment.

(A) Schematic diagram of the experimental set-up for the split-shoot experiment. *Arabidopsis* seedlings were germinated on the growth medium. At d 5, half of the cotyledon was positioned on 40 mM NH_4^+ medium, while the other half of the cotyledon remained in normal growth medium. After 48 h, *EBS:GUS* activity and DAB staining was measured.

(B) Expression of *EBS:GUS* in the split-shoot experiment. One representative sample from each treatment (10 plants) is shown. Scale bars = 1 mm.

(C) DAB staining in the split-shoot experiment. One representative sample from each treatment (10 plants) is shown. Scale bars = 1 mm.

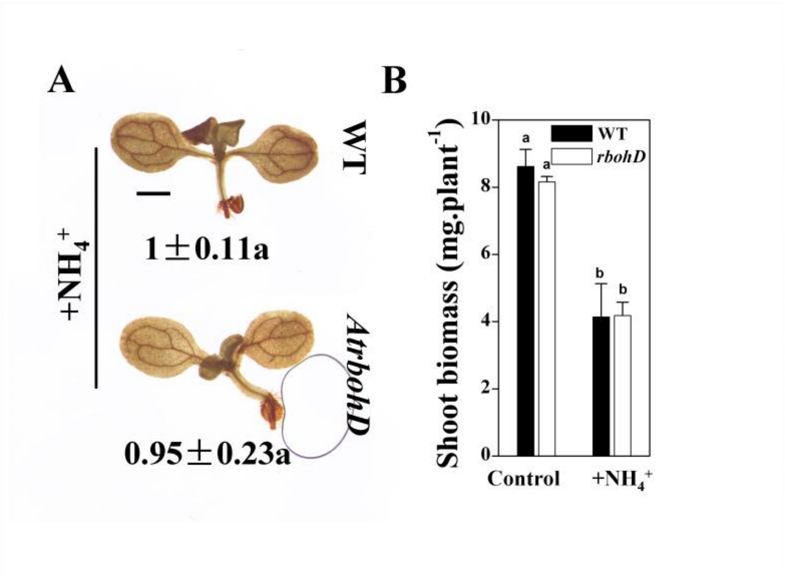

Fig. S6 Effect of NH_4^+ treatment on shoot DAB staining and biomass of WT and the *Atrbohd* mutant.

(A) *In-situ* detection of hydrogen peroxide in leaves of WT and the *Atrbohd* mutant. Seedlings at 5 d were exposed to 40 mM NH_4^+ for 3 d, and then DAB staining of shoots was performed. Scale bars = 1 mm. DAB staining intensity was quantified using Image J software, and the WT was considered as 1. Values are the means ± SD, n = 10. Different letters indicate statistical differences between the mutant and WT (independent samples *t*-test, $*P < 0.05$).

(B) Effect of NH_4^+ treatment on shoot biomass. Seedlings at 5 d were exposed to 40 mM NH_4^+ for 6 d. Values are the mean ± SD, n = 8-11. Different letters indicate statistical differences at $P < 0.05$ (one-way ANOVA analysis with Duncan post-hoc test).

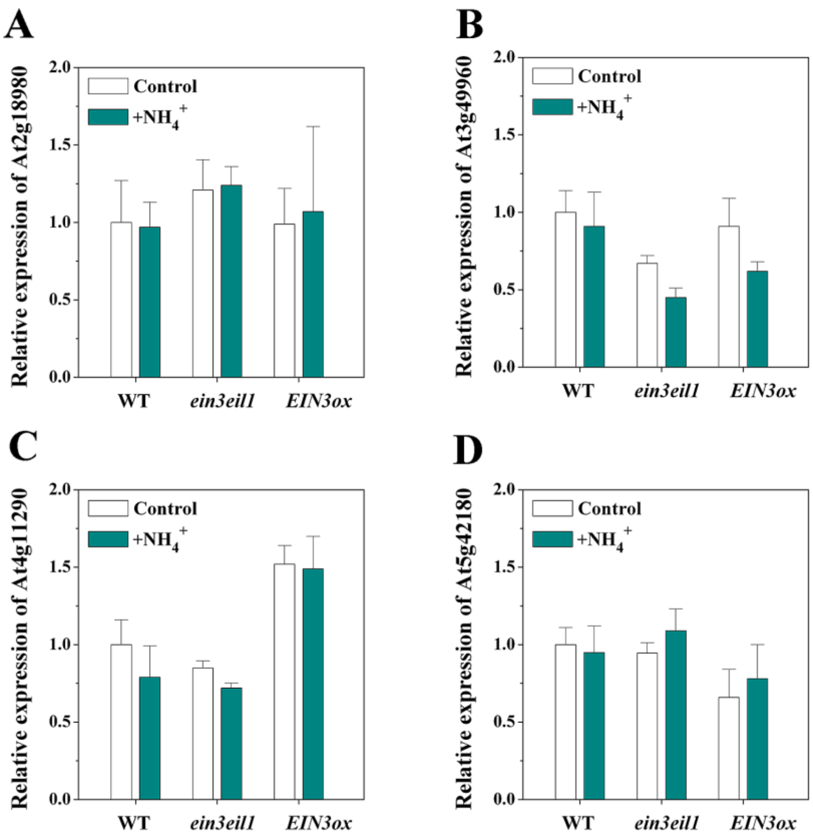

Fig. S7 qRT-PCR analysis of POD gene expression in WT, *EIN3ox*, and *ein3eil1* shoot tissue by quantitative real-time PCR under NH_4^+ treatment for 6 h. Values are means ± SD of three replicates. ACTIN2 was used as the internal reference gene, and the WT control was considered as 1.

Fig. S8 Measurement of POD activity of WT, *EIN3ox,* and *ein3eil1* shoot tissue under control conditions. Seedlings at 5 d were exposed to control medium without NH_4^+ for 5 d. Values are means ± SD of three replicates. Different letters indicate statistical differences at $P < 0.05$ of control and NH_4^+ treatment, respectively (one-way ANOVA analysis with Duncan post-hoc test).

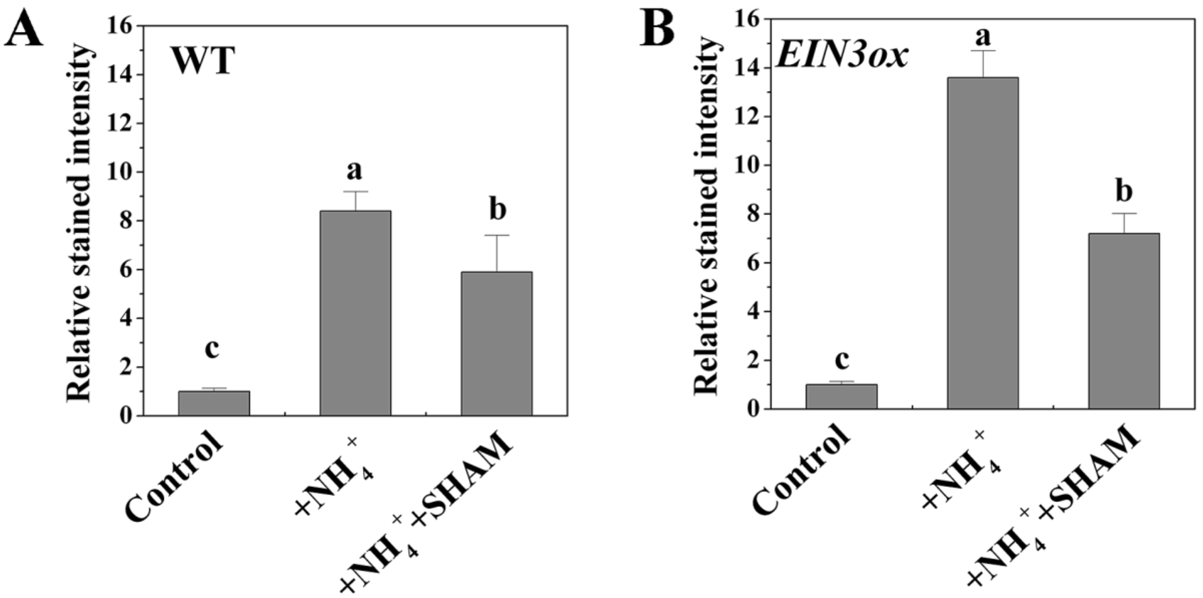

Fig. S9 Mean relative DAB-staining intensity in WT (A) and *EIN3ox* (B) shoots treated with NH_4^+ and NH_4^+ plus SHAM. DAB staining was quantified based on an arbitrary value for the pixel intensity of the stained areas of the leaves in Fig. 7(G). The controls of WT and *EIN3ox* were considered as 1. Values are the means ± SD, n = 7-8. Different letters indicate statistical differences at $P < 0.05$ (one-way ANOVA analysis with Duncan post-hoc test).

Table S1 Gene-specific primers used for qRT-PCR.

Gene	Forward Primers (5'-3')	Reverse Primers (5'-3')
APX1	CTCTGGGACGATGCCACAAG	CTCGACCAAAGGACGGAAAA
APX2	TTGGTCGGATGGGACTCAAT	GAAGAGCCTTGTCGGTTGGT
CAT1	CTGGGATTCAGACAGGCAAGA	AACCAAACCGTAAGAGGAGCAT
CAT2	CCCGTGTCTTCTCCTATGCC	TAACCTCCTCGTCCCTGTGC
CAT3	AATCACAGCCACGCCACTAA	TCAGAACCAAGCGACCAACC
RBOHA	AGGGGTCGTTTGACTGGTTC	CTCGTAAACGCTGGTGCAGT
RBOHB	CGAGGTGATGGGCTACTGTG	TGTTCCTACAAACGGGCAAG
RBOHD	GCTCCGTGCTTTCAGATCAA	TTTGAATCCTTGTGGCTTCG
RBOHF	GCCGACGAAACAACAAAGAA	CACCAATGCCAAGACCAACT
ACS2	TCATGGGAAAAGCTAGAGGTGGAAG	TCAACGGTTAATTTGAAATTGTCGG
ACS7	CCTGGGTTCCGTGAAAACGCATT	CGTCGTTAGGATCGGCGAGAATGA
ACS11	CTGGTTTCGGGTCTAAAGGAAGCGG	AATGACACGATGAGCCTGGAGAGATGTT
ACO2	GGATGTCGGTTGCATCGTTTTA	TACGGCTGCTGTAGGATTCAGTTC
ERF1	CGAGCAGTCCACGCAACAAA	GTCCCGAGCCAAACCCTAAT
EIN3	ACATGGTGGAAGGAAGTT	TTGCCGCTACTGTTATTG
At1g49570	TTGGGACGAAGGGACTCATT	GGGTCACGAACTTGGCTGTT
At5g19890	ATGCGTCGTTATTGTTGGAT	CACAGCGGCTTTGATTGTAT
At2g18980	TCGTCCGTGGATGTGATGCT	CTTTGCCTTTGCCACCGTAT
At3g49960	TTGACTCTAACCCTAGTTGC	TTTGTCTACATTGTCCGAAG
At4g11290	ATGCCGACAATCTCAAATCA	AATGCAGCGTCAGACTCAAA
At5g42180	CCTCCTAACATCTCACTCCA	TTGCCTTTGATATTCTTCCA
CBP20	ACCATCGGAAACGACAAAGAG	CTTCACCATCGTCATCGGAGT
ACTIN2	GCACCCTGTTCTTCTTACCG	AACCCTCGTAGATTGGCACA
